# Holistic Framework to Contextualize Dietary Quality Assessment: A Critical Review

**DOI:** 10.3390/ijerph20053986

**Published:** 2023-02-23

**Authors:** Jessica M. Phelan, Richard R. Rosenkranz, Connor J. Phelan, Sara K. Rosenkranz

**Affiliations:** 1 Department of Food Nutrition Dietetics and Health, Kansas State University, Manhattan, KS 66506, USA; 2 Physical Activity and Nutrition Clinical Research Consortium, Kansas State University, Manhattan, KS 66506, USA; 3 Department of Health and Human Performance, Fort Hays State University, Hays, KS 67601, USA; 4 Department of Kinesiology & Nutrition Sciences, University of Nevada, Las Vegas, NV 89154, USA; 5 Department Geosciences, Fort Hays State University, Hays, KS 67601, USA

**Keywords:** dietary quality, reductionism, holism, dietary quality index, dietary quality measurement, social factors, environmental factors

## Abstract

Numerous dietary quality indices exist to help quantify overall dietary intake and behaviors associated with positive health outcomes. Most indices focus solely on biomedical factors and nutrient or food intake, and exclude the influence of important social and environmental factors associated with dietary intake. Using the Diet Quality Index- International as one sample index to illustrate our proposed holistic conceptual framework, this critical review seeks to elucidate potential adaptations to dietary quality assessment by considering—in parallel—biomedical, environmental, and social factors. Considering these factors would add context to dietary quality assessment, influencing post-assessment recommendations for use across various populations and circumstances. Additionally, individual and population-level evidence-based practices could be informed by contextual social and environmental factors that influence dietary quality to provide more relevant, reasonable, and beneficial nutritional recommendations.

## 1. Introduction

According to the World Health Organization, around 41 million or 71% of annual global deaths are due to noncommunicable diseases (NCD). Diseases that are often related to poor dietary quality and sedentary lifestyles, such as cardiovascular diseases (CVD), respiratory diseases, and diabetes, account for 17.9, 3.9 and 1.6 million deaths, respectively [1]. As the burden of NCDs continues to increase to a greater extent in lower-income countries and populations [2], the importance of understanding the factors that influence dietary quality becomes more pronounced. Dietary quality assessment should consider contextual factors influencing the quality of dietary intake in order to translate such assessments into post-assessment recommendations and potential interventions.

To quantitively determine dietary quality, mathematical algorithms—in the form of dietary quality indices—have been created based upon adherence to pre-defined nutritional guidelines such as the Dietary Guidelines for Americans that are thought to enhance health, support growth, and reduce diet-related diseases [3]. Reviews of the most common indices of dietary quality show that higher scores are associated with a lower incidence of chronic disease. Some studies aimed at reviewing dietary indices, including the Healthy Eating Index (HEI) and the Alternative Healthy Eating Index (AHEI), have shown unclear results regarding specific health outcomes, such as CVD risk factors, risk of developing abdominal obesity, and gender-specific weight loss [4,5]. Other reviews have indicated that better adherence to dietary guidelines is associated with reduced risk of all-cause mortality, CVD mortality, and cancer mortality [6,7]. A meta-analysis assessing adherence to the DASH diet in relation to all-cause and cause-specific mortality suggests more uniform results, where even modest scores are associated with lower all-cause and cause-specific mortality [8].

Waijers et al. [9] suggested that to critically evaluate a dietary quality index, the main questions that need to be considered are why the index was created in the first place, and what health outcome was the index created to address? As mentioned above, the most common indices have been shown to predict health status or risk when dietary quality scores are either high or low. In order to determine the utility of a particular index, however, the purpose behind the creation of the index should be considered [10,11]. Additionally, individual and population-level evidence-based practices should include contextual considerations, such as social and environmental factors. These factors influence the translatability of dietary quality scores for post-assessment nutritional recommendations across differing populations. Such contextual considerations clearly do not alter actual nutrient needs; however, they could be added as metrics for the interpretation of an existing dietary index or used to create entirely new indices. Although as many as 57 major indices of dietary quality exist, the Diet Quality Index-International (DQI-I) is among the most commonly used indexes due to its applicability for international use [12]. The DQI-I was used in the present paper to provide an example index from relevant literature [4,5,12], showing that the DQI-I is appropriate for cross-cultural application.

Using the Giessen Declaration [13] as a guide to broaden the science of nutrition into a more integrative discipline inclusive of the challenges of the twenty-first century, the current critical review seeks to elucidate relevant biomedical, social, and environmental factors in order to foster a more holistic contextual understanding of dietary quality assessment. First, a brief review of the concepts of reductionism and holism will be provided. Next, utilizing the DQI-I as one example index to illustrate this conceptual framework, the current review considers social and environmental factors that contribute to dietary quality in various populations and contexts.

## 2. Reductionism vs. Holism

Historically, nutrition sciences have largely been focused on gaining knowledge about foods and nutrients in relation to health. In 2015, Scrinis wrote “*Nutritionism: The Science and Politics of Dietary Advice*”, a book that summarizes how nutritional advice has changed throughout recent history, with a focus on reductionism [14]. The concept of reductionism can be thought of simply as science that reconstructs reality by its parts [15,16]. With this viewpoint, medicine has approached nutrition in a similar manner to pharmacology, as a treatment for individuals who are unhealthy, rather than as a way to prevent nutrition-related diseases in order to keep people healthy [17]. While reductionism has a history of improving the world’s health—for example, the eradication of scurvy, pellagra, and rickets from all but the poorest of areas—it has also directed scientists to examine the intake of single nutrients or foods, rather than the impact of a pattern of food intake as a whole. For example, antioxidants are known to “decrease oxidative stress”; therefore, many studies have been conducted to determine specifics about their benefits. Many studies have used “supra-physiological” doses of antioxidant vitamins, taken as supplements extrinsic to foods naturally high in vitamin content. Unexpectedly to the researchers, some results have shown an increased risk of cancers at these doses [18]. Other studies have shown that isolated nutrient supplementation may not be beneficial, and relevant health outcomes are more likely to be associated with the overall dietary pattern [19]. There is now an increased recognition of the importance of whole food consumption, and not just individual nutrients, thus introducing the more holistic concept of food synergy [17,20].

Conceptually, food synergy means that in most cases, foods are interactive in their effects on health [20]. Similar to the idea of Gestalt theory, 1 + 1 > 2, or “the whole is more than the sum of its parts [17]”. To illustrate this, in 2018, Fardet, Lakhssassi, and Briffaz conducted a study that provided insights regarding how to classify foods based upon their structures as well as their nutrient compositions, keeping in mind the concept of food synergy [21]. Fardet and colleagues indicated that foods with identical nutrient compositions could have differential effects on health, likely due to the bioavailability of the nutrients in the compared foods as well as their satiating effects; therefore, one calorie of a food did not necessarily equal one calorie of another. For example, 100 calories of two carbohydrate-containing foods, such as jellybeans and grapes, do not provide equal nutrition. In the study, 280 generic foods most widely consumed by French men and women aged 65 years and older were tested to determine how quantitative and qualitative aspects of food, such as texture, water activity, shelf life, glycemic response, and satiety related to levels of food processing. Results showed that minimally processed foods such as cooked lamb, brussels sprouts, hard eggs, broccoli, and pink shrimp, to name a few, were more satiating, more nutritionally dense, less hyperglycemic, had a shorter shelf-life, and in a compression test, had lower maximum stress—meaning they were deformed with less compression than ultra-processed foods [21]. Based on the study results, Fardet and colleagues concluded that foods should first be classified based upon the degree of processing, then nutrient composition, and that food and diet should be looked at as a whole and should include the matrix effect, and then should be looked at according to its component pieces.

## 3. Diet Quality Index-International: Diet Index from a Holistic Perspective

An important aspect to consider in the assessment and translation of dietary quality in practice is measurement: how is overall dietary quality measured? Most dietary indices use records of food consumption over a specified time-period using 24 h recalls, food frequency questionnaires, or daily food logs, and then generate scores indicating high or low adherence to pre-determined population-level dietary guidelines. Dietary indices differ from one to the other, each emphasizing differing aspects of dietary quality. Generally, all indices have been created to emphasize the analysis of the biomedical factors and/or health outcomes associated with food consumption rather than incorporating other social and environmental factors—reflecting the reductionist view. The current review provides an overview of the DQI-I, using it as one sample dietary quality index to illustrate the holistic conceptual framework provided herein. We acknowledge that there are many additional dietary indices that may also be suitable for use.

In order to address the need for a more holistic measurement tool, the Diet Quality Index-International (DQI-I) was developed in 2003 and was used in a cross-national study that compared dietary quality between the United States and China [22]. Kim and colleagues claimed that prior to their study, no cross-national research on dietary quality had been conducted because there was no way to ensure the validity of an existing index for use in a different country or context. Therefore, the reasoning behind creating the DQI-I was to provide a measurement tool for assessing dietary quality that would apply to diverse populations in a comprehensive manner, using the average of two to three 24 h recalls and addressing overnutrition as well as undernutrition. To illustrate this concept, studies from various countries, such as Iran [23], Korea [24], and Canada [25], to name a few, have used the DQI-I as a measurement tool to assess dietary quality and have investigated associations between DQI-I scores and various outcomes, such as obesity and cardiovascular diseases in adults and body fat among children.

The major constructs assessed within the DQI-I are variety, adequacy, moderation, and overall dietary balance. These categories are included based upon the understanding that both foods and individual nutrients are valuable aspects of dietary quality when comparing heterogeneous dietary cultures. Kim and colleagues [22] stated that other previously developed indices often combined both adequacy and moderation into one category, making it difficult to determine the cause of a low score and, more specifically, the impact of a dietary component on an outcome of interest, as shown in Table 1.

As an example of how under or overnutrition may both lead to low overall dietary quality scores, a country with substantial undernutrition in its population could have a low score due to insufficient food sources, thereby indicating poor adequacy. On the other hand, a country known for overnutrition could have a low DQI-I score due to the overconsumption of food, indicating poor moderation. However, both countries would potentially have low overall dietary quality scores if adequacy and moderation were combined into one category, and the reason behind the score would be uncertain. Further information about the DQI-I, its components, and its scoring may be found in the original publication by Kim and colleagues [22].

## 4. Holistic Conceptual Framework for Dietary Quality Assessment

Utilizing the DQI-I as one sample dietary quality index, this section of the review elucidates how social and environmental factors related to dietary quality would add important context for the improved translation of research into practice. Examples of the biomedical, social, and environmental factors in the holistic conceptual framework are shown in Table 2. Additionally, selected sub-factors are discussed in further detail that potentially add novel and current contextual considerations. Well-established factors, such as religion, culture, climate, seasonality, and natural phenomena, remain relevant but are not the focus of this manuscript. Although sub-factors are grouped into social and environmental categories, we acknowledge that many of the sub-factors are multi-faceted and do cross into other factors and sub-factors. Further, biomedical factors will not be discussed in detail in this review, as they are typically already discussed within dietary quality index considerations.

### 4.1. Social Factors

Social factors are integral to the relationship that human beings have with food and are critical to our understanding of food intake behaviors. Food is interwoven into social events, such as weddings and parties, and it is part of religious celebrations, such as Lent and Ramadan [26]. For example, Eastern Orthodox Christian Church members abstain from eating olive oil, meat, fish, eggs and dairy every Wednesday and Friday, and they employ four fasting periods throughout the year [27]. Jewish people have specific food laws (Kosher) and dietary regulations that they abide by [28,29]. For spiritual purposes, Buddhists avoid eating specific vegetables, alcohol, and all animal products except milk [30]. Food is a sign of love and compassion in some cultures; in others, food is a reminder of economic status [31]. Social factors influence food choice and dietary consumption, thereby affecting dietary quality.

### 4.2. Socioeconomics and Food Processing

Although the proportion of people living in poverty has never been lower than it is now, economic disparities throughout the world are as prevalent today as they were 100 years ago. According to the Food and Agriculture Organization of the United Nations, moderate to severe food insecurity affects around 2 billion people worldwide, and from that total, 1.04 billion are in Asia, 676 million are in Africa, and 188 million are in Latin America [32]. Conversely, the obesity epidemic has continued to worsen and has now surpassed 650 million cases in adults worldwide [33]. Economics influence dietary quality and food choice drastically, often times influencing individuals to rely on ultra-processed foods or no food at all rather than fresh, less processed food choices, affecting adequate micronutrient intake [34,35]. Sometimes, ultra-processed foods are cheaper, have a longer shelf life, and are more easily obtained than fresh, nutrient-dense foods. These factors not only make eating nutritious meals more costly and burdensome, but also make obtaining all essential nutrients difficult for segments of populations, or entire populations.

Socioeconomic factors have influenced food consumption throughout history because, in some instances, food has been a symbol of social status. Historically, the ability to purchase a plethora of food coupled with the possibility of surplus led to greater food consumption among the wealthy, resulting in increased body weight. Excess body weight was a sign of wealth and beauty [36]. Today, over-consumption of food has been linked to negative health outcomes and increased risk for non-communicable diseases, but prestige-seeking behaviors are still exhibited in food selection, regardless of nutritional value [37]. Wealthier people purchase and consume prestigious foods perceived to be a symbol of social status, and people with a lower socioeconomic status seek to purchase these same foods in order to be seen as members of as wealthier class, regardless of nutritional value [37]. This is just one example of how social factors influence food consumption and dietary quality.

### 4.3. Environmental Factors

Environmental factors, such as climate, season, natural phenomena, food safety, land availability, rainfall, sunlight, topography, and food accessibility—to name a few—influence dietary quality substantially, especially in developing countries. Organizations such as the FAO have been established to help increase food accessibility and safety around the world through the implementation of sustainable food and agriculture missions, food systems, and more [38]. Both developing and developed countries also face many environmental challenges that influence the adequacy of nutrition intake.

#### 4.3.1. Food Accessibility and Land Availability

According to the FAO, in 2018, around 26.4% of people globally were facing food insecurity [32]. Moderate food insecurity is defined as “having reduced quality and/or quantity of food and uncertainty about the ability to obtain food due to lack of money or other resources”. Severe food insecurity is defined as “having run out of food, and at the most extreme, having gone days without eating”. Socioeconomics are involved in the attainment of food, as well as geographical location in relation to sources of food. Mass food production favors the distribution of food products to areas where larger profits may be obtained, thereby neglecting the distribution of foods to lower-income areas [39]. Urban sprawl contributes to the increase in the distance between food sources and populations. Generally, as cities expand outward into suburban areas, grocery stores and food sources follow. Land availability is scarce in inner-city neighborhoods, and space is tight, discouraging businesses to build restaurants, grocery stores and super-markets. The relocation of food supply stores is also due, at least in part, to wealthier populations often occupying suburban areas. Therefore, inner-city neighborhoods, particularly low-income areas without access to transportation, are devoid of the local food sources, thereby creating food deserts [40]. Food deserts are identified as areas with limited access to affordable and nutritious foods [41]. The determination of food deserts varies but usually take into consideration whether the area is urban or rural, the cost of transportation, the price of foods, the distance to the nearest food store, the number of food stores in the area, types of food offered (fresh or preserved) and the nutritional value of the offered foods [42]. Without close access to food sources, populations located within the food deserts rely on automobile transportation or public transportation. As a result, individuals living in these areas generally consume highly processed foods rather than fresh foods, based upon the expense and availability of the product [43].

#### 4.3.2. Pollution, Contamination, and Food Safety

Pollution, contamination, and food safety are critical factors for populations in all areas of the world. Many countries have advanced regulations regarding sanitation, water treatment, food handling, food production and food regulation. In these areas, measures are taken not only to purify water sources, but also to add different key nutrients to the water supply for the purpose of enhancing health by providing essential micronutrients [44]. Other countries struggle with supplying adequate clean water and safe food to support human life. Without important regulations, foodborne illnesses and diseases run rampant, resulting in sickness, disabilities, and often early mortality [45]. In 2010, an estimated 2 billion illnesses and 1.09 million deaths worldwide were reported from 22 foodborne bacterial, protozoal, and viral diseases, and many likely go unreported [45].

Another important consideration is the effect of pollution. Plastic build-up spans all oceans and seas, potentially harming wildlife and seafood sources. Microplastics found inside whole, sea-dwelling creatures provide the potential for toxicities in humans who consume them [46]. Crop injury, diminished growth, and reduced agricultural yields are present when agriculture is exposed to toxic air pollutants [47]. Agriculturally polluted run-off infiltrates lakes and rivers. Run-off chemicals can have a drastic effect on native fauna, leading to an array of health issues for people that consume them [48]. For example, as a result of intense agricultural practices on the banks of the Racoon River in Central Iowa, nearly three-quarters of the 1.7 million-acre watershed is cultivated. This process requires millions of pounds of fertilizer, pesticides, and other chemicals. Consequently, the rising nitrate level has passed the Environmental Protection Agency’s (EPA) legal limit for drinking water [49]. These factors contribute to food and water availability by decreasing the amount of clean drinking water and safe food production. Without a proper supply of agricultural products, the adequacy of plant and animal-based foods may not meet population needs, thereby jeopardizing adequacy and balance.

## 5. DQI-I: Contextual Translation of Dietary Quality Scores

**Figure 1:**
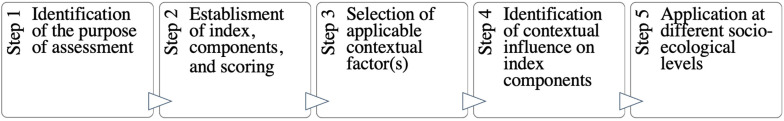
Holistic Dietary Quality Assessment Process.

Some of the most popular dietary indices, such as the HEI, AHEI, and DASH Score indices, are most relevant to high-income countries that have access to plentiful food sources and food imports; therefore, these indices may not be relatable or applicable for low-income countries that struggle with food security [3]. The DQI-I is an index that allows users to customize measurements based upon country-specific guidelines. This customizability provides a foundation for additional considerations that could include other social and environmental contextual factors to provide a more holistic assessment. The DQI-I is used as a focused example index within this critical review, specifically because of its applicability to various contexts. Other indices could also be modified using the conceptual framework suggested to incorporate other holistic factors. The process of how contextual considerations could be incorporated into dietary quality assessment through alterations, exclusions, modifications, and additions to the DQI-I are shown in Figure 1: Holistic Dietary Quality Assessment Process.

The first step in the conceptual Holistic Dietary Quality Assessment Process is to identify the purpose or goal of the assessment to provide direction for the contextualization of overall dietary quality and how the assessment might be applied. Once the goal of the assessment has been identified, the next step is to select the most appropriate dietary index, including its components and scoring methods. For the examples we have provided, we used the DQI-I due to its applicability to different populations and settings [22] where this assessment process might be used [50]. The next step is to select a contextual factor or factors that are thought to potentially influence overall dietary quality. The factors listed in the assessment process model are nowhere near exhaustive of the biomedical, social, or environmental factors that could be present in a population. Additionally, it is understood that these factors are highly interconnected. Once the contextual factors have been identified for measurement, a critical analysis of each index component should be conducted to determine what aspects of dietary quality may be influenced by the selected contextual factor, whether that be variety, adequacy, moderation, balance, or another index component. In other words, what components of the dietary index are being negatively affected due to the additional social or environmental factors. These considerations may add valuable context for evidence-based practices, allowing for the translation of dietary quality scores to the appropriate post-assessment recommendations based on the social and environmental factors that influence dietary quality.

**Figure 2:**
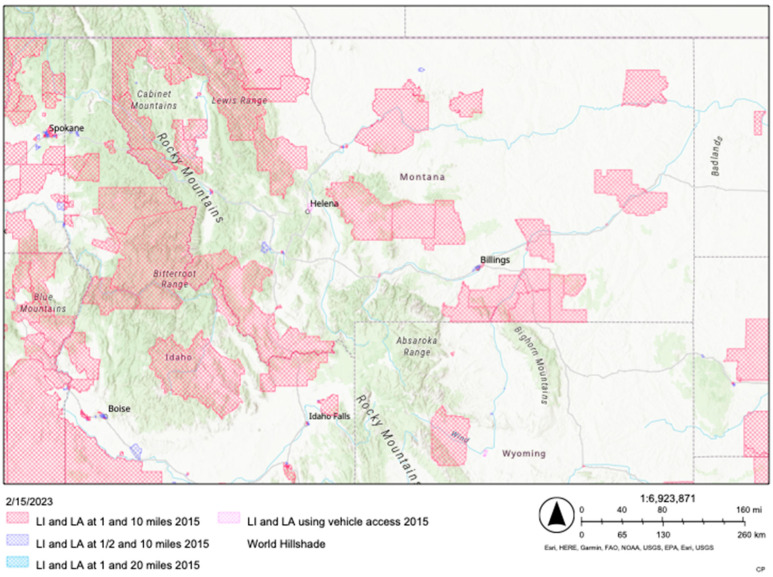
Example of the Food Access Research Atlas Low-Income (LI) Low-Access (LI) Montana.

The following examples are hypothetical illustrations of how this conceptual assessment process might be use in different settings, specifically at the individual and community or societal levels of influence on health [50,51]. Table 3: Holistic Dietary Quality Assessment Process, Example 1: Community or Societal level illustrates an example of a hypothetical community leader identifying potential reasons for poor dietary quality in their respective geographic location. Specifically, we highlight rural areas where accessibility to healthful foods might be lacking and how programming or policy changes might help to improve dietary quality at the community level. In order to determine accessibility, the United States Department of Agriculture: Economic Research Service’s “Food Data Atlas” represents one way to identify areas of low income and areas of low accessibility to healthful foods, as seen in Figure 2: Example of the Food Access Research Atlas [52]. After completing the assessment process steps, the community leader could then determine what feasible, immediate, or minor changes could be made to increase the accessibility of the healthful foods in their community—for example, reducing sales taxes on fresh produce or creating pop-up farmers markets within low-income, low-accessibility areas.

Figure 2 shows a rural location in Montana, USA. Low-income (LI) and Low-access (LA) at 1–10 miles, ½–10 miles, 1–20 miles, and using vehicle access are displayed.

To further demonstrate the assessment process, Table 4: Holistic Dietary Quality Assessment Process, Example 2: Individual level illustrates a hypothetical practitioner helping an individual client who has lactose intolerance and is living in a northern geographic area during winter. Through the identification of barriers to achieving high dietary quality such as decreased Vitamin D intake, the practitioner may focus on strategies to help increase dietary quality, such as switching to lactose-free dairy products, taking a Vitamin D supplement, or focusing on fortified foods. Additionally, by adding contextual factors, the practitioner may identify a “ceiling” for the dietary quality that the client could potentially achieve, considering the factors that are in or out of that client’s control. This process could help to determine potential facilitators to improving dietary quality, making achievable recommendations to increase overall dietary quality. The goal would be able to maximize the potential dietary quality, recognizing that some factors would not be controllable and may not be easily modified.

## 6. Cross-Population Comparisons

The flexibility for dietary quality scoring to be based on country-specific guidelines, when used within the context of a larger, uniform scale, provides a foundation for a holistic conceptual framework for evaluation across different cultures and populations. A more holistic and useful analysis of dietary quality may be possible when evaluation of dietary quality (1) includes contextual, social and environmental factors according to country or region based on their specific dietary guidelines or the Food and Agriculture Organization of the United Nations (FAO) guidelines, and (2) considers variety, adequacy, moderation, and balance. Further, this more contextualized and holistic approach may allow insights into the explanatory mechanisms behind dietary quality differences than are typically gleaned from assessments of dietary quality. For example, if a study were to evaluate the dietary quality of high-income countries (e.g., Canada) or populations where there is access to a large variety of imported foods versus populations from a desert-climate low-income country (e.g., Libya) with few imported food products, dietary intakes and guidelines would likely differ drastically. While it is possible that indices of dietary quality could be modified to add biomedical, social, and environmental factors in order to provide a more holistic assessment, another potential approach would be to utilize previously designed dietary quality indices with minor modifications alongside separate tools that assess social and environmental factors anytime dietary quality is assessed. For example, if a practitioner suspects that a social or environmental factor may be a barrier to achieving high dietary quality scores, this conceptual framework may be used to determine what contextual factors are present, thereby allowing alterations to post-assessment recommendations and potential interventions.

## 7. Conclusions

The purpose of the current critical review was not to offer a definitive solution for the problem of poor dietary quality, but rather to shine a light on the need to further inquire about how we might accomplish the inclusion of contextual factors when seeking to understand the barriers and facilitators for optimizing dietary quality. Considering contextual factors alongside adherence to dietary guidelines in dietary quality assessment could strengthen the core of evidence-based practices. Evidence-based practices are essential for providing high-quality care, guiding the development of policies, determining preventive practices, and much more. An improved understanding of individual and population-level goals, expectations, preferences, accessibility, and resources through evidence-based nutrition practices will produce reasonable, applicable, and beneficial nutritional recommendations. A holistic approach to dietary quality assessment may allow nutrition professionals to better identify areas for improvement, such as potential barriers contributing to poor adherence to guidelines within dietary intervention studies, or specific population needs for overcoming barriers to positive health outcomes. If dietary quality is substantially hindered due to social or environmental factors, public health officials could use these data to work toward mitigating barriers to the extent possible. Further, the identification of the populations that are doing well with regard to dietary quality, even while facing social or environmental challenges, could assist nutrition scientists in advising government agency professionals developing future dietary guidelines.

## Figures and Tables

**Table 1:** Comparison of Index Scoring Components: Adequacy and Moderation.

Dietary Quality Index-International Separate Scoring of Moderation and Adequacy					Other Index Combined Scoring of Moderation and Adequacy			
Undernutrition	→	Low adequacy score	→	Low overall Dietary quality	←	Low adequacy OR moderation score	←	Undernutrition
Overnutrition	→	Low moderation score	→				←	Overnutrition

**Table 2:** Holistic Factors Contributing to Translation of Dietary Quality.

Holistic Factors Contributing to Contextual Translation of Dietary Quality		
Biomedical Factors	Social Factors	Environmental Factors
Health/Disease Status	Culture	Climate/Seasons
Genetics	Socioeconomics	Pollution/Contamination
Anthropometrics	Religion	Land Availability
Energy Balance	Food Preparation	Food Accessibility
Developmental Status	Food Processing	Food Safety/Regulation
Personal Attributes	Meal Patterns	Topography

**Table 3:** Holistic Dietary Quality Assessment Process, Example 1: Community or Societal level.

Holistic Dietary Quality Assessment Process				
Example 1: Community or Societal-level				
Step 1: Identification of Index, Components, and Scoring	Step 2: Selection of Applicable Contextual Factor/Factors	Step 3: Identification of Contextual Influence on Index Component		
**Variety: (0–20 pts)** Overall variety (0–15 pts) Within-group (0–5 pts)	**Biomedical** Health/Disease Status Genetics Anthropometrics Energy Balance Developmental Status Personal Attributes **Social** Culture Socioeconomics Religion Food Preparation Food Processing Meal Patterns **Environmental** Climate/Seasons Pollution/Contamination Land Availability *Food Accessibility* Food Safety/Regulation Topography	**Variety** (0–20 pts)	→	The “within-group” variety for protein might be limited due to not including points for other protein sources such as grains, legumes, seeds, soy, or other geographic-specific protein sources.
**Adequacy: (0–40 pts)** Vegetable (0–5 pts) Fruit (0–5 pts) Grain (0–5 pts) Fiber (0–5 pts) Protein (0–5 pts) Iron (0–5 pts) Calcium (0–5 pts) Vitamin C (0–5 pts)		**Adequacy** (0–40 pts)	→	Different types of nutritional data, such as a 24 h recall, might alter the accuracy of this component due to accessibility issues differing from one day to another. A FFQ might be more appropriate during this context, as it allows the dietary quality score to analyze intake over a period of time.
**Moderation: (0–30 pts)** Total Fat (0–6 pts) Saturated Fat (0–6 pts) Cholesterol (0–6 pts) Sodium (0–6 pts) Empty Calories (0–6 pts)		**Moderation** (0–30 pts)	→	Ready-to-eat foods or highly processed foods might be eaten more regularly due to difficulties in accessing fresh produce. These foods are often high in saturated fats, sugars, sodium, and empty calories.
**Balance: (0–10 pts)** Macronutrient ratio (0–6 pts) Fatty acid ratio (0–4 pts)		**Balance** (0–10 pts)	→	The types of foods potentially consumed when fresh produce is not readily available, such as foods with a long shelf-life, could contribute to an off-balance fatty acid ratio.

Bold font denotes headings of major index scoring areas and contextual factor groups. Italic font denotes the contextual factor/s identified in the assessment process.

**Table 4:** Conceptual Holistic Dietary Quality Assessment Process, Example 2: Individual level.

Holistic Dietary Quality Assessment Process				
Example 2: Individual-Level				
Step 1:Identification of Index, Components, and Scoring	Step 2: Selection of Applicable Contextual Factor/Factors	Step 3: Identification of Contextual Influence on Index Component		
**Variety: (0–20 pts)** Overall variety (0–15 pts) Within–group (0–5 pts)	**Biomedical** *Health/Disease Status* Genetics Anthropometrics Energy Balance Developmental Status Personal Attributes **Social** Culture Socioeconomics Religion Food Preparation Food Processing Meal Patterns **Environmental** *Climate/Seasons* Pollution/Contamination Land Availability Food Accessibility Food Safety/Regulation Topography	**Variety** (0–20 pts)	→	Variety score might be low due to low accessibility to in-season fruits and vegetables.
**Adequacy: (0–40 pts)** Vegetable (0–5 pts) Fruit (0–5 pts) Grain (0–5 pts) Fiber (0–5 pts) Protein (0–5 pts) Iron (0–5 pts) Calcium (0–5 pts) Vitamin C (0–5 pts)		**Adequacy** (0–40 pts)	→	Special consideration might be given to foods that are plentiful sources of Vitamin D.
**Moderation: (0–30 pts)** Total Fat (0–6 pts) Saturated Fat (0–6 pts) Cholesterol (0–6 pts) Sodium (0–6 pts) Empty Calories (0–6 pts)		**Moderation** (0–30 pts)	→	Some of the foods that would still contribute to increased health could also contain high amounts of sodium, such as canned vegetables.
**Balance: (0–10 pts)** Macronutrient ratio (0–6 pts) Fatty acid ratio (0–4 pts)		**Balance** (0–10 pts)	→	Ratios could be off-balance if the person is unable to consume specific foods.

Bold font denotes headings of major index scoring areas and contextual factor groups. Italic font denotes the contextual factor/s identified in the assessment process.

## Data Availability

Data sharing not applicable.
